# Effects of Animal-Assisted Therapy on Verbal Communication and Agitation in Adults With Dementia in Long-Term Care

**DOI:** 10.1093/geroni/igaa057.032

**Published:** 2020-12-16

**Authors:** Jenilee Woltman, Ladan Ghazi Saidi

**Affiliations:** University of Nebraska Kearney, Kearney, Nebraska, United States

## Abstract

The effects of Animal-Assisted Therapy (AAT) on verbal communication and agitated behaviors of older adults with mild to severe dementia were examined. We conducted a pilot study at a long-term nursing facility in rural Nebraska on five residents with various stages of dementia and agitation (over two reported incidents of agitation within two months). The study consisted of four sessions of assessment with and four sessions without the presence of a certified therapy dog. The assessment battery included a Mean Length of Utterance (MLU), and responding to a Picture Recognition Activity (PRA) task to assess the production and comprehension in verbal communication respectively. Agitation Behavioral Scale (ABS; Corrigan, 1989), and a General Care Survey (GCS) were used to measure agitation and agitated behavior. The results showed statistically significant differences in the performance of all five participants when the dog was present as compared to when it was absent. We observed an increase in verbal communication (57.4% MLU; 28% PRA), and a decrease in agitated behaviors. This pilot study brings evidence that AAT can be a cost-effective, non-pharmaceutical therapy approach for individuals with dementia to decrease agitation and increase communication. References: Corrigan, J. D. 1989. Development of a scale for assessment of agitation following traumatic brain injury. Journal of Clinical and Experimental Neuropsychology, 11: 261–277.

